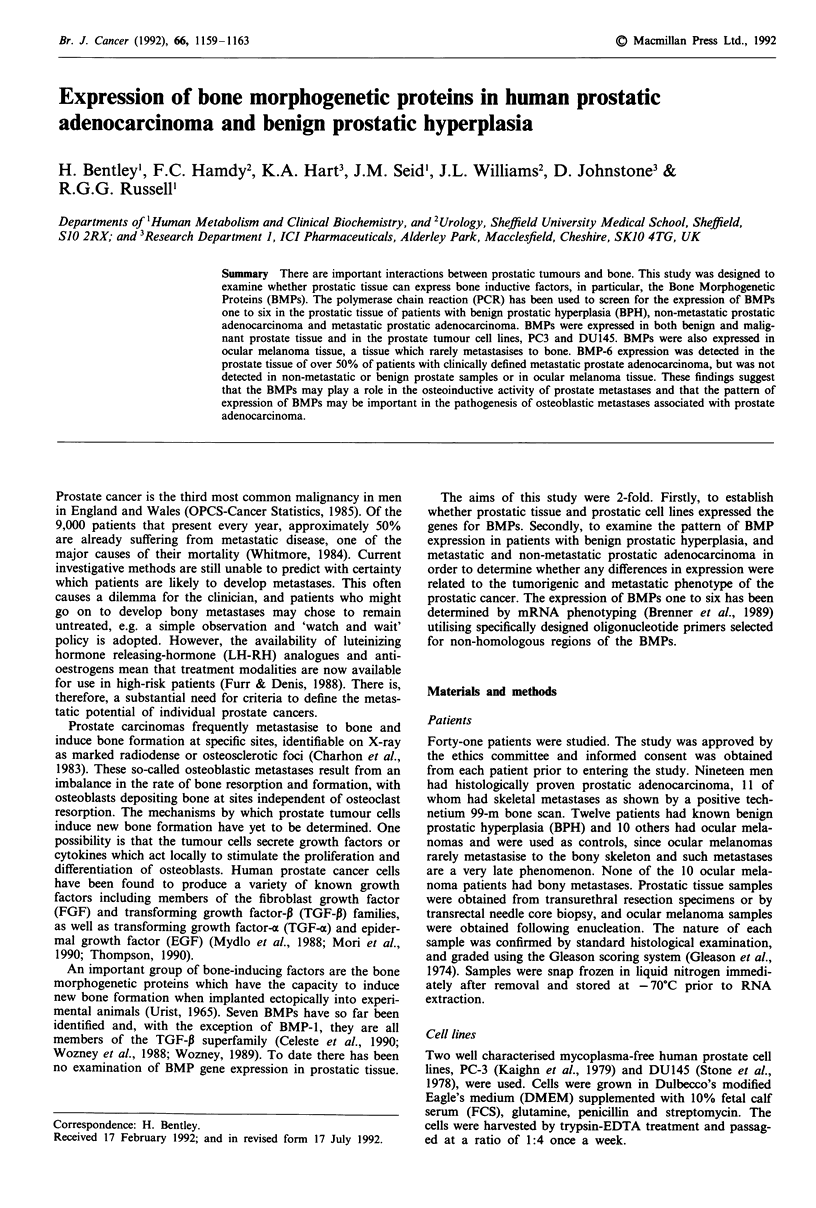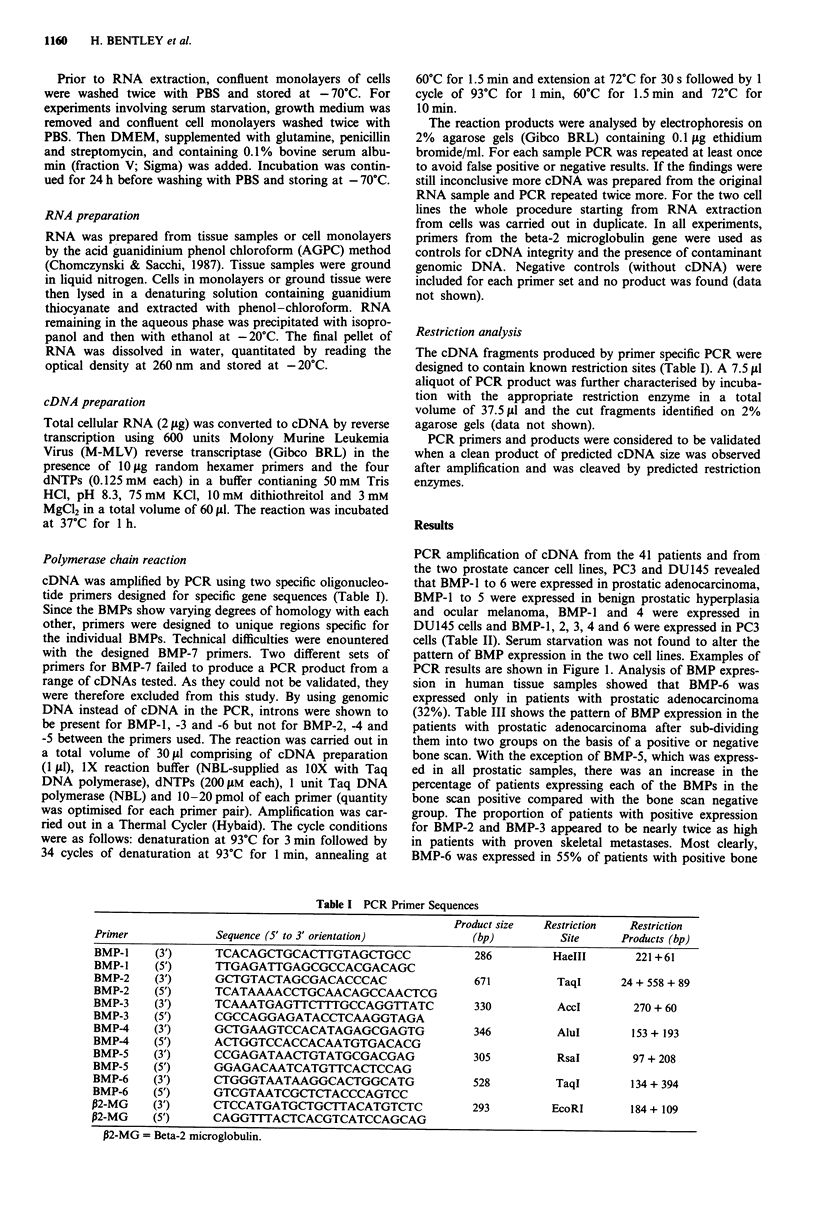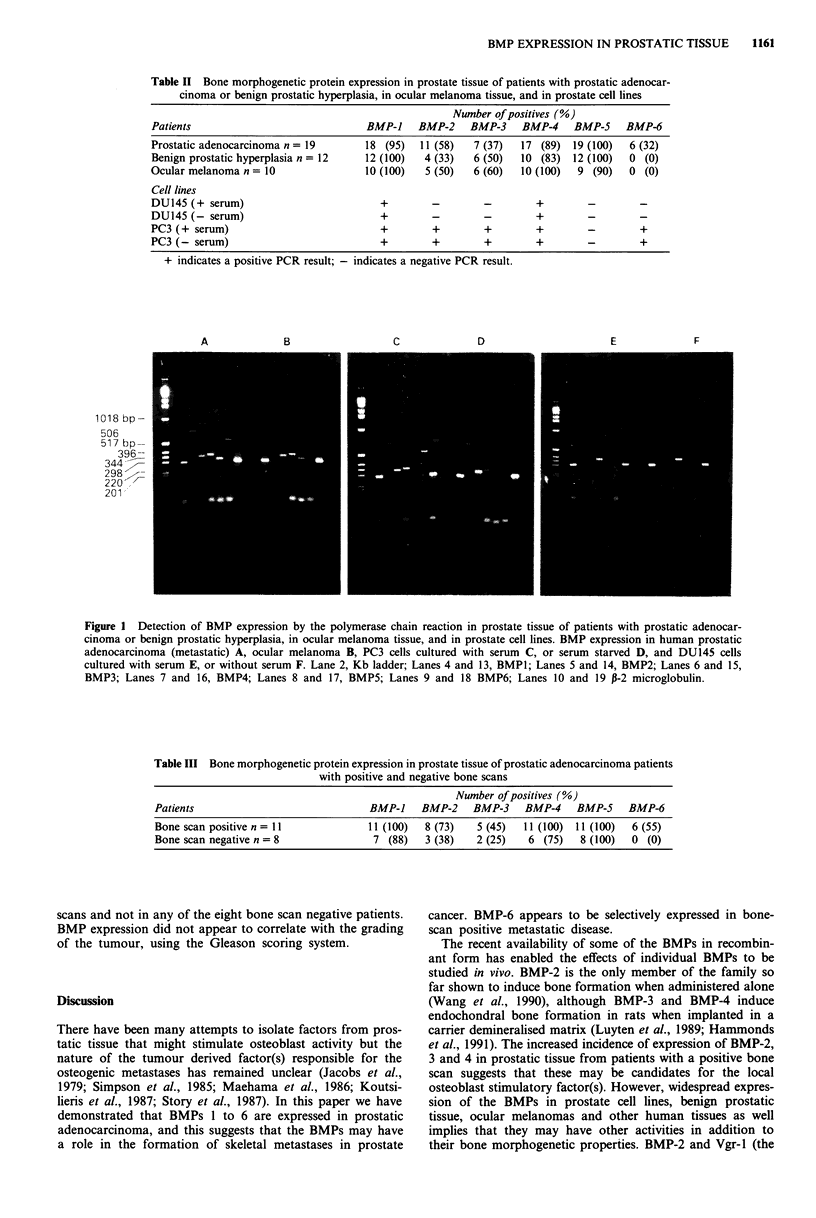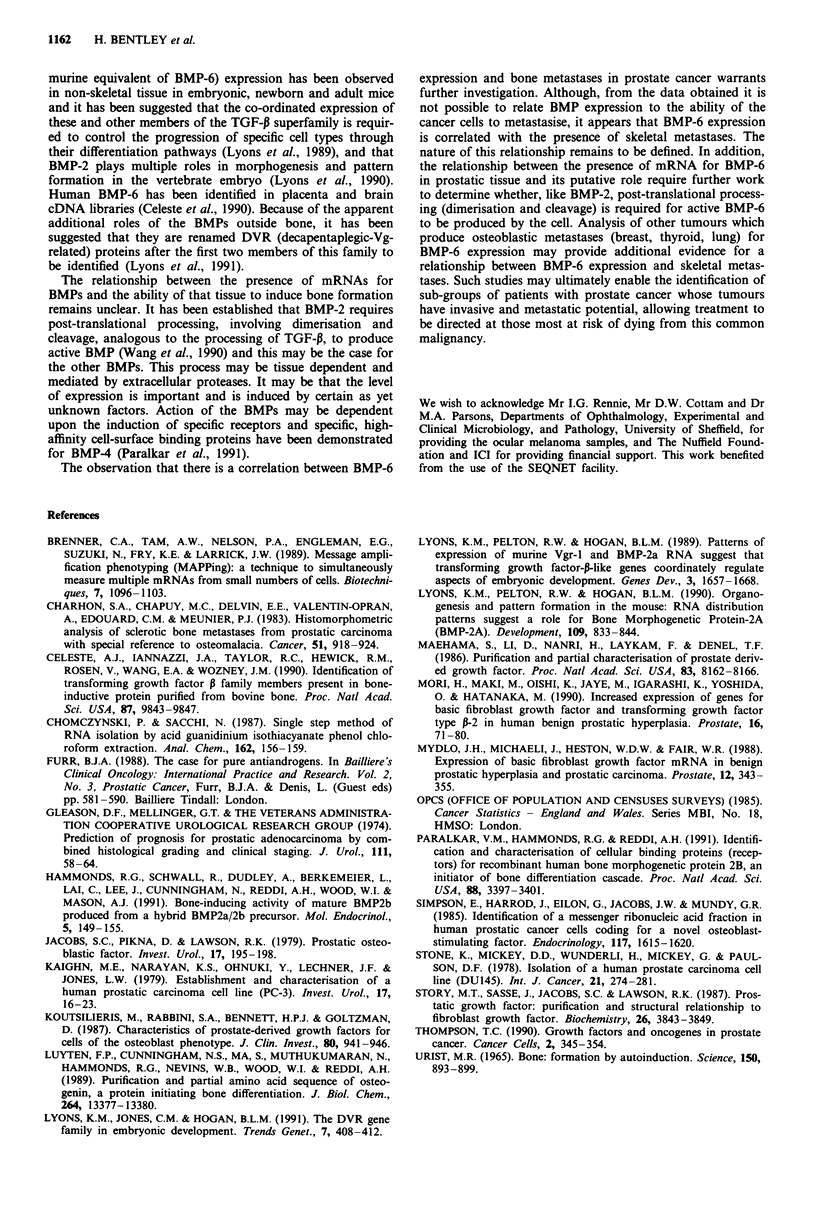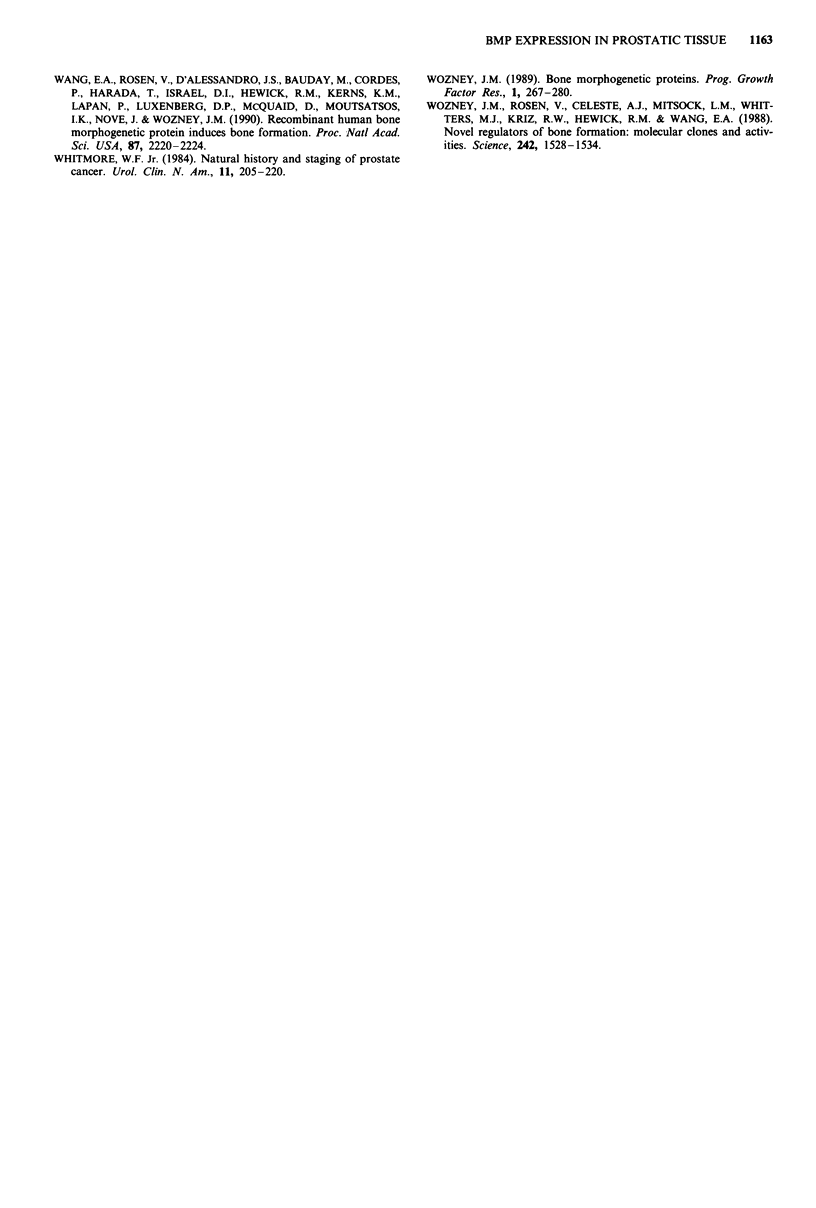# Expression of bone morphogenetic proteins in human prostatic adenocarcinoma and benign prostatic hyperplasia.

**DOI:** 10.1038/bjc.1992.427

**Published:** 1992-12

**Authors:** H. Bentley, F. C. Hamdy, K. A. Hart, J. M. Seid, J. L. Williams, D. Johnstone, R. G. Russell

**Affiliations:** Department of Human Metabolism and Clinical Biochemistry, Sheffield University Medical School, UK.

## Abstract

**Images:**


Br. J. Cancer (1992), 66, 1159-1163 © Macmillan Press Ltd., 1992

### Expression of bone morphogenetic proteins in human prostatic adenocarcinoma and benign prostatic hyperplasia

H. Bentley[1], F.C. Hamdy[2], K.A. Hart[3], J.M. Seid[1], J.L. Williams[2], D. Johnstone[3] & R.G.G. Russell[1]

*Departments of [1]Human Metabolism and Clinical Biochemistry, and [2]Urology, Sheffield University Medical School, Sheffield, S10 2RX; and [3]Research Department 1, ICI Pharmaceuticals, Alderley Park, Macclesfield, Cheshire, SK10 4TG, UK*

**Summary** There are important interactions between prostatic tumours and bone. This study was designed to examine whether prostatic tissue can express bone inductive factors, in particular, the Bone Morphogenetic Proteins (BMPs). The polymerase chain reaction (PCR) has been used to screen for the expression of BMPs one to six in the prostatic tissue of patients with benign prostatic hyperplasia (BPH), non-metastatic prostatic adenocarcinoma and metastatic prostatic adenocarcinoma. BMPs were expressed in both benign and malignant prostate tissue and in the prostate tumour cell lines, PC3 and DU145. BMPs were also expressed in ocular melanoma tissue, a tissue which rarely metastasises to bone. BMP-6 expression was detected in the prostate tissue of over 50% of patients with clinically defined metastatic prostate adenocarcinoma, but was not detected in non-metastatic or benign prostate samples or in ocular melanoma tissue. These findings suggest that the BMPs may play a role in the osteoinductive activity of prostate metastases and that the pattern of expression of BMPs may be important in the pathogenesis of osteoblastic metastases associated with prostate adenocarcinoma.

Prostate cancer is the third most common malignancy in men in England and Wales (OPCS-Cancer Statistics, 1985). Of the 9,000 patients that present every year, approximately 50% are already suffering from metastatic disease, one of the major causes of their mortality (Whitmore, 1984). Current investigative methods are still unable to predict with certainty which patients are likely to develop metastases. This often causes a dilemma for the clinician, and patients who might go on to develop bony metastases may chose to remain untreated, e.g. a simple observation and 'watch and wait' policy is adopted. However, the availability of luteinizing hormone releasing-hormone (LH-RH) analogues and anti-oestrogens mean that treatment modalities are now available for use in high-risk patients (Furr & Denis, 1988). There is, therefore, a substantial need for criteria to define the metastatic potential of individual prostate cancers.

Prostate carcinomas frequently metastasise to bone and induce bone formation at specific sites, identifiable on X-ray as marked radiodense or osteosclerotic foci (Charhon *et al.*, 1983). These so-called osteoblastic metastases result from an imbalance in the rate of bone resorption and formation, with osteoblasts depositing bone at sites independent of osteoclast resorption. The mechanisms by which prostate tumour cells induce new bone formation have yet to be determined. One possibility is that the tumour cells secrete growth factors or cytokines which act locally to stimulate the proliferation and differentiation of osteoblasts. Human prostate cancer cells have been found to produce a variety of known growth factors including members of the fibroblast growth factor (FGF) and transforming growth factor-β (TGF-β) families, as well as transforming growth factor-α (TGF-α) and epidermal growth factor (EGF) (Mydlo *et al.*, 1988; Mori *et al.*, 1990; Thompson, 1990).

An important group of bone-inducing factors are the bone morphogenetic proteins which have the capacity to induce new bone formation when implanted ectopically into experimental animals (Urist, 1965). Seven BMPs have so far been identified and, with the exception of BMP-1, they are all members of the TGF-β superfamily (Celeste *et al.*, 1990; Wozney *et al.*, 1988; Wozney, 1989). To date there has been no examination of BMP gene expression in prostatic tissue.

Correspondence: H. Bentley.
Received 17 February 1992; and in revised form 17 July 1992.

The aims of this study were 2-fold. Firstly, to establish whether prostatic tissue and prostatic cell lines expressed the genes for BMPs. Secondly, to examine the pattern of BMP expression in patients with benign prostatic hyperplasia, and metastatic and non-metastatic prostatic adenocarcinoma in order to determine whether any differences in expression were related to the tumorigenic and metastatic phenotype of the prostatic cancer. The expression of BMPs one to six has been determined by mRNA phenotyping (Brenner *et al.*, 1989) utilising specifically designed oligonucleotide primers selected for non-homologous regions of the BMPs.

### Materials and methods

### *Patients*

Forty-one patients were studied. The study was approved by the ethics committee and informed consent was obtained from each patient prior to entering the study. Nineteen men had histologically proven prostatic adenocarcinoma, 11 of whom had skeletal metastases as shown by a positive technetium 99-m bone scan. Twelve patients had known benign prostatic hyperplasia (BPH) and 10 others had ocular melanomas and were used as controls, since ocular melanomas rarely metastasise to the bony skeleton and such metastases are a very late phenomenon. None of the 10 ocular melanoma patients had bony metastases. Prostatic tissue samples were obtained from transurethral resection specimens or by transrectal needle core biopsy, and ocular melanoma samples were obtained following enucleation. The nature of each sample was confirmed by standard histological examination, and graded using the Gleason scoring system (Gleason *et al.*, 1974). Samples were snap frozen in liquid nitrogen immediately after removal and stored at −70°C prior to RNA extraction.

### *Cell lines*

Two well characterised mycoplasma-free human prostate cell lines, PC-3 (Kaighn *et al.*, 1979) and DU145 (Stone *et al.*, 1978), were used. Cells were grown in Dulbecco's modified Eagle's medium (DMEM) supplemented with 10% fetal calf serum (FCS), glutamine, penicillin and streptomycin. The cells were harvested by trypsin-EDTA treatment and passaged at a ratio of 1:4 once a week.

Prior to RNA extraction, confluent monolayers of cells were washed twice with PBS and stored at −70°C. For experiments involving serum starvation, growth medium was removed and confluent cell monolayers washed twice with PBS. Then DMEM, supplemented with glutamine, penicillin and streptomycin, and containing 0.1% bovine serum albumin (fraction V; Sigma) was added. Incubation was continued for 24 h before washing with PBS and storing at −70°C.

*RNA preparation*

RNA was prepared from tissue samples or cell monolayers by the acid guanidinium phenol chloroform (AGPC) method (Chomczynski & Sacchi, 1987). Tissue samples were ground in liquid nitrogen. Cells in monolayers or ground tissue were then lysed in a denaturing solution containing guanidium thiocyanate and extracted with phenol–chloroform. RNA remaining in the aqueous phase was precipitated with isopropanol and then with ethanol at −20°C. The final pellet of RNA was dissolved in water, quantitated by reading the optical density at 260 nm and stored at −20°C.

*cDNA preparation*

Total cellular RNA (2 μg) was converted to cDNA by reverse transcription using 600 units Molony Murine Leukemia Virus (M-MLV) reverse transcriptase (Gibco BRL) in the presence of 10 μg random hexamer primers and the four dNTPs (0.125 mM each) in a buffer contianing 50 mM Tris HCl, pH 8.3, 75 mM KCl, 10 mM dithiothreitol and 3 mM $MgCl_2$ in a total volume of 60 μl. The reaction was incubated at 37°C for 1 h.

*Polymerase chain reaction*

cDNA was amplified by PCR using two specific oligonucleotide primers designed for specific gene sequences (Table I). Since the BMPs show varying degrees of homology with each other, primers were designed to unique regions specific for the individual BMPs. Technical difficulties were enountered with the designed BMP-7 primers. Two different sets of primers for BMP-7 failed to produce a PCR product from a range of cDNAs tested. As they could not be validated, they were therefore excluded from this study. By using genomic DNA instead of cDNA in the PCR, introns were shown to be present for BMP-1, -3 and -6 but not for BMP-2, -4 and -5 between the primers used. The reaction was carried out in a total volume of 30 μl comprising of cDNA preparation (1 μl), 1X reaction buffer (NBL-supplied as 10X with Taq DNA polymerase), dNTPs (200 μM each), 1 unit Taq DNA polymerase (NBL) and 10–20 pmol of each primer (quantity was optimised for each primer pair). Amplification was carried out in a Thermal Cycler (Hybaid). The cycle conditions were as follows: denaturation at 93°C for 3 min followed by 34 cycles of denaturation at 93°C for 1 min, annealing at 60°C for 1.5 min and extension at 72°C for 30 s followed by 1 cycle of 93°C for 1 min, 60°C for 1.5 min and 72°C for 10 min.

The reaction products were analysed by electrophoresis on 2% agarose gels (Gibco BRL) containing 0.1 μg ethidium bromide/ml. For each sample PCR was repeated at least once to avoid false positive or negative results. If the findings were still inconclusive more cDNA was prepared from the original RNA sample and PCR repeated twice more. For the two cell lines the whole procedure starting from RNA extraction from cells was carried out in duplicate. In all experiments, primers from the beta-2 microglobulin gene were used as controls for cDNA integrity and the presence of contaminant genomic DNA. Negative controls (without cDNA) were included for each primer set and no product was found (data not shown).

*Restriction analysis*

The cDNA fragments produced by primer specific PCR were designed to contain known restriction sites (Table I). A 7.5 μl aliquot of PCR product was further characterised by incubation with the appropriate restriction enzyme in a total volume of 37.5 μl and the cut fragments identified on 2% agarose gels (data not shown).

PCR primers and products were considered to be validated when a clean product of predicted cDNA size was observed after amplification and was cleaved by predicted restriction enzymes.

### Results

PCR amplification of cDNA from the 41 patients and from the two prostate cancer cell lines, PC3 and DU145 revealed that BMP-1 to 6 were expressed in prostatic adenocarcinoma, BMP-1 to 5 were expressed in benign prostatic hyperplasia and ocular melanoma, BMP-1 and 4 were expressed in DU145 cells and BMP-1, 2, 3, 4 and 6 were expressed in PC3 cells (Table II). Serum starvation was not found to alter the pattern of BMP expression in the two cell lines. Examples of PCR results are shown in Figure 1. Analysis of BMP expression in human tissue samples showed that BMP-6 was expressed only in patients with prostatic adenocarcinoma (32%). Table III shows the pattern of BMP expression in the patients with prostatic adenocarcinoma after sub-dividing them into two groups on the basis of a positive or negative bone scan. With the exception of BMP-5, which was expressed in all prostatic samples, there was an increase in the percentage of patients expressing each of the BMPs in the bone scan positive compared with the bone scan negative group. The proportion of patients with positive expression for BMP-2 and BMP-3 appeared to be nearly twice as high in patients with proven skeletal metastases. Most clearly, BMP-6 was expressed in 55% of patients with positive bone

**Table I** PCR Primer Sequences

| *Primer* | | *Sequence (5′ to 3′ orientation)* | *Product size (bp)* | *Restriction Site* | *Restriction Products (bp)* |
|---|---|---|---|---|---|
| BMP-1 | (3′) | TCACAGCTGCACTTGTAGCTGCC | 286 | HaeIII | 221 + 61 |
| BMP-1 | (5′) | TTGAGATTGAGCGCCACGACAGC | | | |
| BMP-2 | (3′) | GCTGTACTAGCGACACCCAC | 671 | TaqI | 24 + 558 + 89 |
| BMP-2 | (5′) | TCATAAAACCTGCAACAGCCAACTCG | | | |
| BMP-3 | (3′) | TCAAATGAGTTCTTTGCCAGGTTATC | 330 | AccI | 270 + 60 |
| BMP-3 | (5′) | CGCCAGGAGATACCTCAAGGTAGA | | | |
| BMP-4 | (3′) | GCTGAAGTCCACATAGAGCGAGTG | 346 | AluI | 153 + 193 |
| BMP-4 | (5′) | ACTGGTCCACCACAATGTGACACG | | | |
| BMP-5 | (3′) | CCGAGATAACTGTATGCGACGAG | 305 | RsaI | 97 + 208 |
| BMP-5 | (5′) | GGAGACAATCATGTTCACTCCAG | | | |
| BMP-6 | (3′) | CTGGGTAATAAGGCACTGGCATG | 528 | TaqI | 134 + 394 |
| BMP-6 | (5′) | GTCGTAATCGCTCTACCCAGTCC | | | |
| β2-MG | (3′) | CTCCATGATGCTGCTTACATGTCTC | 293 | EcoRI | 184 + 109 |
| β2-MG | (5′) | CAGGTTTACTCACGTCATCCAGCAG | | | |

β2-MG = Beta-2 microglobulin.

**Table II** Bone morphogenetic protein expression in prostate tissue of patients with prostatic adenocarcinoma or benign prostatic hyperplasia, in ocular melanoma tissue, and in prostate cell lines

| | *Number of positives (%)* | | | | | |
|---|---|---|---|---|---|---|
| *Patients* | *BMP-1* | *BMP-2* | *BMP-3* | *BMP-4* | *BMP-5* | *BMP-6* |
| Prostatic adenocarcinoma $n = 19$ | 18 (95) | 11 (58) | 7 (37) | 17 (89) | 19 (100) | 6 (32) |
| Benign prostatic hyperplasia $n = 12$ | 12 (100) | 4 (33) | 6 (50) | 10 (83) | 12 (100) | 0 (0) |
| Ocular melanoma $n = 10$ | 10 (100) | 5 (50) | 6 (60) | 10 (100) | 9 (90) | 0 (0) |
| *Cell lines* | | | | | | |
| DU145 (+ serum) | + | – | – | + | – | – |
| DU145 (– serum) | + | – | – | + | – | – |
| PC3 (+ serum) | + | + | + | + | – | + |
| PC3 (– serum) | + | + | + | + | – | + |

+ indicates a positive PCR result; – indicates a negative PCR result.

**Figure 1** Detection of BMP expression by the polymerase chain reaction in prostate tissue of patients with prostatic adenocarcinoma or benign prostatic hyperplasia, in ocular melanoma tissue, and in prostate cell lines. BMP expression in human prostatic adenocarcinoma (metastatic) **A**, ocular melanoma **B**, PC3 cells cultured with serum **C**, or serum starved **D**, and DU145 cells cultured with serum **E**, or without serum **F**. Lane 2, Kb ladder; Lanes 4 and 13, BMP1; Lanes 5 and 14, BMP2; Lanes 6 and 15, BMP3; Lanes 7 and 16, BMP4; Lanes 8 and 17, BMP5; Lanes 9 and 18 BMP6; Lanes 10 and 19 $\beta$-2 microglobulin.

**Table III** Bone morphogenetic protein expression in prostate tissue of prostatic adenocarcinoma patients with positive and negative bone scans

| | *Number of positives (%)* | | | | | |
|---|---|---|---|---|---|---|
| *Patients* | *BMP-1* | *BMP-2* | *BMP-3* | *BMP-4* | *BMP-5* | *BMP-6* |
| Bone scan positive $n = 11$ | 11 (100) | 8 (73) | 5 (45) | 11 (100) | 11 (100) | 6 (55) |
| Bone scan negative $n = 8$ | 7 (88) | 3 (38) | 2 (25) | 6 (75) | 8 (100) | 0 (0) |

scans and not in any of the eight bone scan negative patients. BMP expression did not appear to correlate with the grading of the tumour, using the Gleason scoring system.

### Discussion

There have been many attempts to isolate factors from prostatic tissue that might stimulate osteoblast activity but the nature of the tumour derived factor(s) responsible for the osteogenic metastases has remained unclear (Jacobs *et al.*, 1979; Simpson *et al.*, 1985; Maehama *et al.*, 1986; Koutsilieris *et al.*, 1987; Story *et al.*, 1987). In this paper we have demonstrated that BMPs 1 to 6 are expressed in prostatic adenocarcinoma, and this suggests that the BMPs may have a role in the formation of skeletal metastases in prostate cancer. BMP-6 appears to be selectively expressed in bone-scan positive metastatic disease.

The recent availability of some of the BMPs in recombinant form has enabled the effects of individual BMPs to be studied *in vivo*. BMP-2 is the only member of the family so far shown to induce bone formation when administered alone (Wang *et al.*, 1990), although BMP-3 and BMP-4 induce endochondral bone formation in rats when implanted in a carrier demineralised matrix (Luyten *et al.*, 1989; Hammonds *et al.*, 1991). The increased incidence of expression of BMP-2, 3 and 4 in prostatic tissue from patients with a positive bone scan suggests that these may be candidates for the local osteoblast stimulatory factor(s). However, widespread expression of the BMPs in prostate cell lines, benign prostatic tissue, ocular melanomas and other human tissues as well implies that they may have other activities in addition to their bone morphogenetic properties. BMP-2 and Vgr-1 (the murine equivalent of BMP-6) expression has been observed in non-skeletal tissue in embryonic, newborn and adult mice and it has been suggested that the co-ordinated expression of these and other members of the TGF-$\beta$ superfamily is required to control the progression of specific cell types through their differentiation pathways (Lyons *et al.*, 1989), and that BMP-2 plays multiple roles in morphogenesis and pattern formation in the vertebrate embryo (Lyons *et al.*, 1990). Human BMP-6 has been identified in placenta and brain cDNA libraries (Celeste *et al.*, 1990). Because of the apparent additional roles of the BMPs outside bone, it has been suggested that they are renamed DVR (decapentaplegic-Vg-related) proteins after the first two members of this family to be identified (Lyons *et al.*, 1991).

The relationship between the presence of mRNAs for BMPs and the ability of that tissue to induce bone formation remains unclear. It has been established that BMP-2 requires post-translational processing, involving dimerisation and cleavage, analogous to the processing of TGF-$\beta$, to produce active BMP (Wang *et al.*, 1990) and this may be the case for the other BMPs. This process may be tissue dependent and mediated by extracellular proteases. It may be that the level of expression is important and is induced by certain as yet unknown factors. Action of the BMPs may be dependent upon the induction of specific receptors and specific, high-affinity cell-surface binding proteins have been demonstrated for BMP-4 (Paralkar *et al.*, 1991).

The observation that there is a correlation between BMP-6 expression and bone metastases in prostate cancer warrants further investigation. Although, from the data obtained it is not possible to relate BMP expression to the ability of the cancer cells to metastasise, it appears that BMP-6 expression is correlated with the presence of skeletal metastases. The nature of this relationship remains to be defined. In addition, the relationship between the presence of mRNA for BMP-6 in prostatic tissue and its putative role require further work to determine whether, like BMP-2, post-translational processing (dimerisation and cleavage) is required for active BMP-6 to be produced by the cell. Analysis of other tumours which produce osteoblastic metastases (breast, thyroid, lung) for BMP-6 expression may provide additional evidence for a relationship between BMP-6 expression and skeletal metastases. Such studies may ultimately enable the identification of sub-groups of patients with prostate cancer whose tumours have invasive and metastatic potential, allowing treatment to be directed at those most at risk of dying from this common malignancy.

We wish to acknowledge Mr I.G. Rennie, Mr D.W. Cottam and Dr M.A. Parsons, Departments of Ophthalmology, Experimental and Clinical Microbiology, and Pathology, University of Sheffield, for providing the ocular melanoma samples, and The Nuffield Foundation and ICI for providing financial support. This work benefited from the use of the SEQNET facility.

### References

BRENNER, C.A., TAM, A.W., NELSON, P.A., ENGLEMAN, E.G., SUZUKI, N., FRY, K.E. & LARRICK, J.W. (1989). Message amplification phenotyping (MAPPing): a technique to simultaneously measure multiple mRNAs from small numbers of cells. *Biotechniques*, **7**, 1096–1103.

CHARHON, S.A., CHAPUY, M.C., DELVIN, E.E., VALENTIN-OPRAN, A., EDOUARD, C.M. & MEUNIER, P.J. (1983). Histomorphometric analysis of sclerotic bone metastases from prostatic carcinoma with special reference to osteomalacia. *Cancer*, **51**, 918–924.

CELESTE, A.J., IANNAZZI, J.A., TAYLOR, R.C., HEWICK, R.M., ROSEN, V., WANG, E.A. & WOZNEY, J.M. (1990). Identification of transforming growth factor $\beta$ family members present in bone-inductive protein purified from bovine bone. *Proc. Natl Acad. Sci. USA*, **87**, 9843–9847.

CHOMCZYNSKI, P. & SACCHI, N. (1987). Single step method of RNA isolation by acid guanidinium isothiacyanate phenol chloroform extraction. *Anal. Chem.*, **162**, 156–159.

FURR, B.J.A. (1988). The case for pure antiandrogens. In *Bailliere's Clinical Oncology: International Practice and Research. Vol. 2, No. 3, Prostatic Cancer*, Furr, B.J.A. & Denis, L. (Guest eds) pp. 581–590. Bailliere Tindall: London.

GLEASON, D.F., MELLINGER, G.T. & THE VETERANS ADMINISTRATION COOPERATIVE UROLOGICAL RESEARCH GROUP (1974). Prediction of prognosis for prostatic adenocarcinoma by combined histological grading and clinical staging. *J. Urol.*, **111**, 58–64.

HAMMONDS, R.G., SCHWALL, R., DUDLEY, A., BERKEMEIER, L., LAI, C., LEE, J., CUNNINGHAM, N., REDDI, A.H., WOOD, W.I. & MASON, A.J. (1991). Bone-inducing activity of mature BMP2b produced from a hybrid BMP2a/2b precursor. *Mol. Endocrinol.*, **5**, 149–155.

JACOBS, S.C., PIKNA, D. & LAWSON, R.K. (1979). Prostatic osteoblastic factor. *Invest. Urol.*, **17**, 195–198.

KAIGHN, M.E., NARAYAN, K.S., OHNUKI, Y., LECHNER, J.F. & JONES, L.W. (1979). Establishment and characterisation of a human prostatic carcinoma cell line (PC-3). *Invest. Urol.*, **17**, 16–23.

KOUTSILIERIS, M., RABBINI, S.A., BENNETT, H.P.J. & GOLTZMAN, D. (1987). Characteristics of prostate-derived growth factors for cells of the osteoblast phenotype. *J. Clin. Invest.*, **80**, 941–946.

LUYTEN, F.P., CUNNINGHAM, N.S., MA, S., MUTHUKUMARAN, N., HAMMONDS, R.G., NEVINS, W.B., WOOD, W.I. & REDDI, A.H. (1989). Purification and partial amino acid sequence of osteogenin, a protein initiating bone differentiation. *J. Biol. Chem.*, **264**, 13377–13380.

LYONS, K.M., JONES, C.M. & HOGAN, B.L.M. (1991). The DVR gene family in embryonic development. *Trends Genet.*, **7**, 408–412.

LYONS, K.M., PELTON, R.W. & HOGAN, B.L.M. (1989). Patterns of expression of murine Vgr-1 and BMP-2a RNA suggest that transforming growth factor-$\beta$-like genes coordinately regulate aspects of embryonic development. *Genes Dev.*, **3**, 1657–1668.

LYONS, K.M., PELTON, R.W. & HOGAN, B.L.M. (1990). Organogenesis and pattern formation in the mouse: RNA distribution patterns suggest a role for Bone Morphogenetic Protein-2A (BMP-2A). *Development*, **109**, 833–844.

MAEHAMA, S., LI, D., NANRI, H., LAYKAM, F. & DENEL, T.F. (1986). Purification and partial characterisation of prostate derived growth factor. *Proc. Natl Acad. Sci. USA*, **83**, 8162–8166.

MORI, H., MAKI, M., OISHI, K., JAYE, M., IGARASHI, K., YOSHIDA, O. & HATANAKA, M. (1990). Increased expression of genes for basic fibroblast growth factor and transforming growth factor type $\beta$-2 in human benign prostatic hyperplasia. *Prostate*, **16**, 71–80.

MYDLO, J.H., MICHAELI, J., HESTON, W.D.W. & FAIR, W.R. (1988). Expression of basic fibroblast growth factor mRNA in benign prostatic hyperplasia and prostatic carcinoma. *Prostate*, **12**, 343–355.

OPCS (OFFICE OF POPULATION AND CENSUSES SURVEYS) (1985). *Cancer Statistics – England and Wales*. Series MBI, No. 18, HMSO: London.

PARALKAR, V.M., HAMMONDS, R.G. & REDDI, A.H. (1991). Identification and characterisation of cellular binding proteins (receptors) for recombinant human bone morphogenetic protein 2B, an initiator of bone differentiation cascade. *Proc. Natl Acad. Sci. USA*, **88**, 3397–3401.

SIMPSON, E., HARROD, J., EILON, G., JACOBS, J.W. & MUNDY, G.R. (1985). Identification of a messenger ribonucleic acid fraction in human prostatic cancer cells coding for a novel osteoblast-stimulating factor. *Endocrinology*, **117**, 1615–1620.

STONE, K., MICKEY, D.D., WUNDERLI, H., MICKEY, G. & PAULSON, D.F. (1978). Isolation of a human prostate carcinoma cell line (DU145). *Int. J. Cancer*, **21**, 274–281.

STORY, M.T., SASSE, J., JACOBS, S.C. & LAWSON, R.K. (1987). Prostatic growth factor: purification and structural relationship to fibroblast growth factor. *Biochemistry*, **26**, 3843–3849.

THOMPSON, T.C. (1990). Growth factors and oncogenes in prostate cancer. *Cancer Cells*, **2**, 345–354.

URIST, M.R. (1965). Bone: formation by autoinduction. *Science*, **150**, 893–899.

WANG, E.A., ROSEN, V., D'ALESSANDRO, J.S., BAUDAY, M., CORDES, P., HARADA, T., ISRAEL, D.I., HEWICK, R.M., KERNS, K.M., LAPAN, P., LUXENBERG, D.P., MCQUAID, D., MOUTSATSOS, I.K., NOVE, J. & WOZNEY, J.M. (1990). Recombinant human bone morphogenetic protein induces bone formation. *Proc. Natl Acad. Sci. USA*, **87**, 2220-2224.

WHITMORE, W.F. Jr. (1984). Natural history and staging of prostate cancer. *Urol. Clin. N. Am.*, **11**, 205-220.

WOZNEY, J.M. (1989). Bone morphogenetic proteins. *Prog. Growth Factor Res.*, **1**, 267-280.

WOZNEY, J.M., ROSEN, V., CELESTE, A.J., MITSOCK, L.M., WHITTERS, M.J., KRIZ, R.W., HEWICK, R.M. & WANG, E.A. (1988). Novel regulators of bone formation: molecular clones and activities. *Science*, **242**, 1528-1534.